# Kiwifruit Non-Sugar Components Reduce Glycaemic Response to Co-Ingested Cereal in Humans

**DOI:** 10.3390/nu9111195

**Published:** 2017-10-30

**Authors:** Suman Mishra, Haley Edwards, Duncan Hedderley, John Podd, John Monro

**Affiliations:** 1 New Zealand Institute for Plant and Food Research, Palmerston North 4442, New Zealand; suman.mishra@plantandfood.co.nz (S.M.); Duncan.hedderley@plantandfood.co.nz (D.H.); 2 Department of Psychology, Massey University, Palmerston North 4442, New Zealand; haley.edwards@massey.ac.nz (H.E.); john.podd@massey.ac.nz (J.P.); 3 Riddet Institute, Massey University, Palmerston North 4442, New Zealand

**Keywords:** kiwifruit, postprandial glycaemia, hypoglycaemia, carbohydrates

## Abstract

Kiwifruit (KF) effects on the human glycaemic response to co-ingested wheat cereal were determined. Participants (*n* = 20) consumed four meals in random order, all being made to 40 g of the same available carbohydrate, by adding kiwifruit sugars (KF sug; glucose, fructose, sucrose 2:2:1) to meals not containing KF. The meals were flaked wheat biscuit (WB)+KFsug, WB+KF, WB+guar gum+KFsug, WB+guar gum+KF, that was ingested after fasting overnight. Blood glucose was monitored 3 h and hunger measured at 180 min post-meal using a visual analogue scale. KF and guar reduced postprandial blood glucose response amplitude, and prevented subsequent hypoglycaemia that occurred with WB+KFsug. The area between the blood glucose response curve and baseline from 0 to 180 min was not significantly different between meals, 0–120 min areas were significantly reduced by KF and/or guar. Area from 120 to 180 min was positive for KF, guar, and KF+guar, while the area for the WB meal was negative. Hunger at 180 min was significantly reduced by KF and/or guar when compared with WB. We conclude that KF components other than available carbohydrate may improve the glycaemic response profile to co-ingested cereal food.

## 1. Introduction

Glucose intolerance is growing in prevalence globally [1,2,3]. It is an important condition to address because of the multiple illnesses that arise as complications of long-term exposure to elevated blood glucose concentrations and of repeated acute postprandial blood glucose excursions [4].

The rate of portal loading of sugars released from the foods by digestion is a dominant net process governing the relative blood glucose response to foods. It is governed by a number of factors that are operating at the gut level. Transfer of digestion products from the digesta to the brush border by mixing, diffusive transfer at the brush border, and absorption, are all rate-limiting steps that modulate the influence of a food on glycaemic response [5,6]. In fruit, factors that may determine the glycaemic impact of sugars include the effect of intactness of fruit tissue particles on sugar release [7], the influence of the fruit tissue and cell wall remnants, including soluble and insoluble cell wall polysaccharides, on gut rheology [8], inhibition of sugar transport at the gut wall by phenolics [9,10,11], and organic acid-induced delay in gastric emptying [12,13].

We have found that ripe kiwifruit disintegrate into a dispersion of cell wall remnants during in vitro digestion. The settled volume occupied by the dispersion is several times greater than the volume of the original fruit; two fruit (200 mL) would yield a dispersion of pulp (800 mL) occupying most of the gastric volume. Within the dispersion, the rate of glucose diffusion and the rate of mixing were each reduced by about 40%, and particles of the dispersion greatly amplified the viscosity of hydrocolloid that was present in the same suspension, with a further reduction in mixing [14].

The ability of kiwifruit cell wall remnants to reduce diffusion and mixing, as well as to interact with soluble fibres in a meal, and thereby alter gut processes involved in the glycaemic response, raised the possibility that kiwifruit consumed with a highly glycaemic food might be able to beneficially alter the glycaemic response to the food. Furthermore, the physical interaction of guar gum with kiwifruit remnants demonstrated in vitro [14] suggested that kiwifruit consumed with hydrocolloids such as guar gum might accentuate the anti-glycaemic influence of hydrocolloids in the diet [6]. Guar has been shown to substantially reduce the amplitude and extend the duration of the postprandial glycaemic response to available carbohydrate. Either a reduced amplitude of the immediate postprandial response and/or a change in the response profile could indicate an improved carbohydrate management by the body, with consequences for downstream states such as hunger and cognition.

Being able to demonstrate that kiwifruit may lower the glycaemic impact of a starchy meal by more than the simple substitution of fructose for starch is important in the context of health claims. Fructose, either free of in sucrose, constitutes about half of the available carbohydrate in kiwifruit, as in many other fruit, and it is disposed of in the liver in obesogenic metabolic pathways. Therefore, although fructose confers low glycaemic potency, there is concern about its possible negative metabolic effects [15], so any health claims for a lowering of glycaemic response by kiwifruit would be likely to be rejected if the effect were due solely to substitution of glucose-yielding carbohydrate by fructose. For instance, a low glycaemic index (GI) claim for a fruit juice was recently rejected because the low GI could not be attributed to more than the fructose content of the drink [15].

To test for a role for non-sugar (non-fructose) components in any glycaemia-moderating effect of kiwifruit we measured changes in blood glucose concentrations for 180 min in response to four meals that contained:

- breakfast cereal plus the sugar loading of kiwifruit,
- breakfast cereal with complete kiwifruit,
- breakfast cereal with guar gum (a hydrocolloid) plus the sugar loading of kiwifruit, and
- and breakfast cereal with guar gum plus complete kiwifruit.

Hunger experienced at 180 min was also measured. The meals were formulated to contain exactly the same type and quantity of available carbohydrate, so that any differences could be attributed to the non-available carbohydrate components of the kiwifruit, to guar gum, or to the interaction of kiwifruit with guar gum. We hypothesised that kiwifruit would cause beneficial changes in the blood glucose response to co-ingested glycaemic cereal product by mechanisms unrelated to the intake and type of sugars in the kiwifruit.

## 2. Materials and Methods

### 2.1. Meal Components

Green kiwifruit (var ‘Hayward’) were obtained from the Plant & Food Research orchard at Te Puke, New Zealand. They were harvested in good condition, and firm but close to ripe. The fruit were stored at room temperature and processed as they ripened, with ripeness being assessed by hand-felt firmness and confirmed by eating. They were peeled and the hard apical core removed, halved, and stored frozen (−20 °C) until enough fruit for the trial had been accumulated over a period of about two weeks. The frozen fruit were allowed to partially thaw and were then crushed to a coarse pulp by briefly (10 s) chopping in a Halde food processor. The pulp was then divided accurately into individual 200 g portions, each stored frozen within a plastic, capped, freezer-proof sundae container.

The breakfast cereal was Weet-Bix™ (WB; Sanitarium, Auckland, New Zealand), which is a whole wheat biscuit that is commonly consumed in Australia and New Zealand and made of cooked wheat flakes compressed into a biscuit that disintegrates rapidly on wetting, and consists mainly of rapidly digestible starch with little sugar (Nutrient information: Protein 12%, Fat 1.4%, Carbohydrate 67% of which sugars 2.8%).

Food-grade guar gum in the form of a fine powder (ISGUAR.5, Ingredient Stop Guar), and fructose were obtained from Hawkins Watts Limited, Penrose, Auckland, New Zealand.

The glucose used was dextrose monohydrate (Davis Food Ingredients, Palmerston North, New Zealand), which contains 91% glucose. It is henceforth referred to as glucose, and allowance was made for its water content in all calculations and weights.

### 2.2. Analysis of Kiwifruit

Subsamples (15 mL) of the kiwifruit pulp were measured accurately into 70 mL specimen pottles in triplicate. They were adjusted to pH 6.5 by titration with 1 M NaOH solution, 50 mL 0.2 M Na maleate buffer (5 mL, pH 6.5) was added, and made to 50 mL with 1% NaCl solution. The pottles were closed, heated to 80 °C for 10 min to gelatinise starch, and cooled to 37 °C before adding 1 mL of 2% pancreatin (Sigma P1750) (Sigma, St. Louis, MO, USA) and 0.1 mL of invertase concentrate (Megazyme E-INVERT) (Megazyme, Bray, Ireland) and the tubes were incubated for 1 h at 30 °C to hydrolyse starch and sucrose to glucose and fructose for analysis. A 1 mL aliquot was removed to a tube containing 4 mL absolute ethanol, mixed and allowed to stand at least 30 min at room temperature before centrifuging (2000 rpm) prior to an analysis of sugar in the supernatant. An invertase-free digestion was also conducted and the difference in reducing sugar between the digestions with and without invertase was used to estimate the sucrose content of the kiwifruit by difference in reducing sugars.

The available carbohydrate content of the digested pulp was measured using a reduced scale modification of the dinitrosalicylic acid (DNS) method [16], and the fructose content was measured by the thiobarbituric acid procedure [17].

### 2.3. Available Carbohydrate Analysis of the Meals

The available carbohydrate content of the meals was measured by a validated in vitro digestion procedure [16], using exactly one tenth of each of the meals in a volume of 50 mL. The samples were moistened with 10 mL of 1% NaCl solution and adjusted to pH 2.5 with 1 M HCl. One millilitre of 10% pepsin (Sigma P-7125) (Sigma, St Louis, MO, USA) solution was added and the pottle incubated at 37 °C for 45 min to simulate gastric digestion. Maleate buffer (5 mL, 0.2 M) was added and the pH adjusted to 6.5 with 0.1 M NaOH. The volume was accurately made to the 53 mL mark and pancreatic digestion commenced by adding 1 mL of 2% pancreatin (Sigma P-1750) solution. Samples (1 mL) were removed at 0, 20, 60, and 120 min into tubes containing 4 mL ethanol to stop the digestion, and the tubes were mixed before storing cold until the sugar analysis. Sugar analyses were conducted as outline above and the results plotted to provide a digestion curve for each meal.

A 2 mL aliquot of the remaining digest was removed from the invertase-containing digests to a 12 mL screw capped tube, 5 mL acetate buffer pH 5.2 added and the tube heated for 20 min in a boiling water bath. Heat-stable amylase (50 μL, Megazyme E-BLAAM) (Megazyme, Bray, Ireland) was added and the tubes mixed and allowed to cool to 40 °C when 50 μL of amyloglucosidase (Megazyme E-AMG) (Megazyme, Bray, Ireland) was added. The tubes were mixed and allowed to stand for 30 min, after which a 1 mL aliquot was removed to 4 mL of ethanol for analysis of total “available” carbohydrate digestion by reducing sugar analysis.

### 2.4. Buffering Capacity of WB+S and WB+KF Meals

The buffering capacity of kiwifruit in the WB+S and WB+KF meals was tested in light of the difference in glycaemic response to the two meals obtained in the present study, in a post hoc titration. The quantities of the meals ingested in a glycaemic response test were each titrated with 0.1 M HCl from their initial pH to pH 2.5, and the titration then continued using 0.1 M NaOH to pH 7.0. The quantities of acid and alkali used were expressed as milliequivalents (mEq).

### 2.5. Formulation of the Meals

Four meals were used in the intervention study, all containing the same quantity of WB and the same quantity and type of sugars as in kiwifruit, added in kiwifruit or as free sugars (S) (Table 1). The meals were formulated to contain 40 g available carbohydrate based on the analysis of the available carbohydrate in the kiwifruit pulp, and on the available carbohydrate value for WB as determined by in vitro digestive analysis.

In meals not containing 200 g of kiwifruit, the kiwifruit was substituted by the amount of sugars that were present in the kiwifruit, so that all of the meals had the same and equal amounts of available carbohydrate. The ratio of the sugars in the kiwifruit was: glucose:fructose:sucrose (2:2:1), so the 18.3 g of available carbohydrate added to the non-kiwifruit meals was made up of 7.32 g glucose plus 7.32 g fructose plus 3.66 g sucrose (Table 2).

The dose of guar gum (10 g) was based on that used by Nilsson, et al. [18] to obtain a reduced glycaemic response by feeding subjects 179 g bread made from flour that had been 15% substituted by guar gum. To ensure that all of the meals in the preset study were of almost equal volume 180 mL of water was consumed with meals not containing kiwifruit.

### 2.6. Human Intervention Study

**Figure 1:**
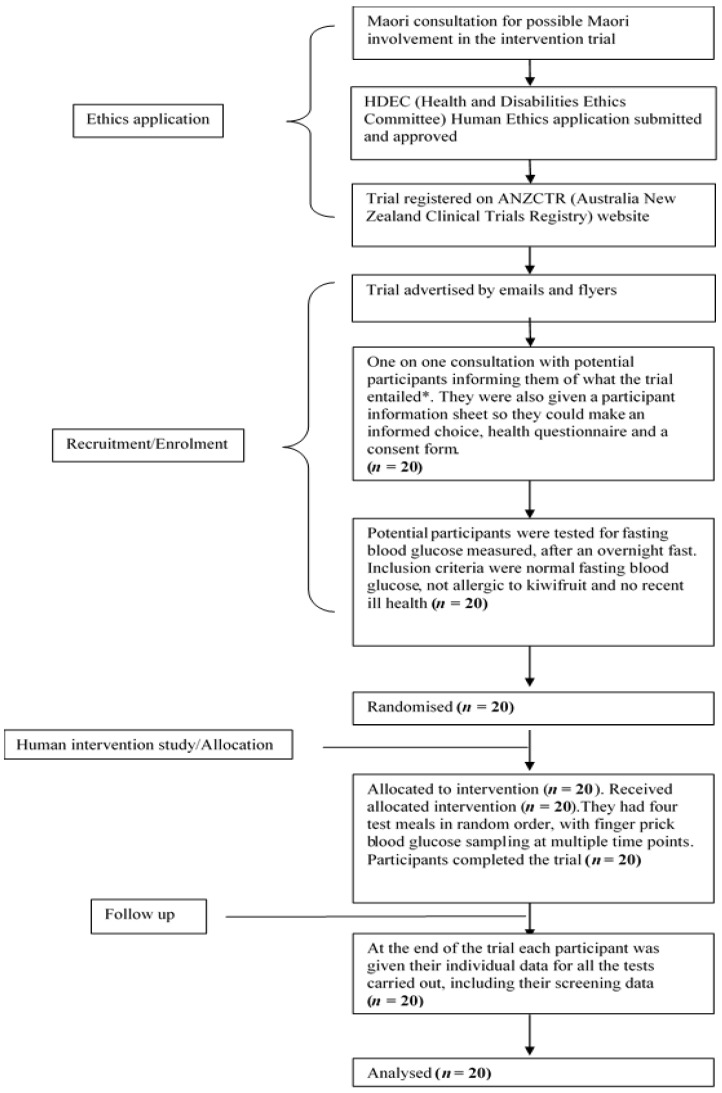
Participant flow chart shows ethical approval, recruitment and intervention processes for the trial. * The participants were allowed to bring a family member of their “Whanau” (support person, family or friend) as family support is highly valued in Maori culture.

The human intervention study was approved by the Human and Disabilities Ethics Committee of the New Zealand Ministry of Health (Ethics number 14/STH/77), and the trial was registered with the Australia New Zealand Clinical Trials Registry (Trial ID: ACTRN12615001259538). The participant flow chart shows ethical approval, recruitment, and intervention processes for the trial, as shown in Figure 1. The CONSORT checklist is attached as Supplementary Table S1. A written informed consent was obtained from participants.

The trial was run as a non-blinded randomised repeated measures study. It was not possible to blind the subjects to the meals they were consuming. However, the data and statistical analysis was performed by an analyst who was blinded to the treatments.

### 2.7. Subjects

Twenty subjects, 6 male and 14 female between the ages of 26 and 66, with a mean age of 36 were recruited by flyer and email. Subject numbers were based on similar published trials, comparing glycaemic responses to foods, and the number was confirmed as adequate by the results of this study. Respondents were interviewed and given an information pack including a description of the study and a consent form. Prospective participants were asked to complete a health questionnaire. Exclusion criteria included known intolerance of kiwifruit, glucose intolerance, and recent ill health.

### 2.8. Preparation of Meals

The dry ingredients for each meal were thoroughly mixed. The moist component (kiwifruit pulp or 180 mL water) was then added with rapid mixing to prevent the formation of lumps. The meals were consumed with 200 mL water in addition to that within the meal.

### 2.9. Glycaemic Response

Subjects were allowed to consume their customary diet but were asked to fast overnight for at least 12 h and present themselves at 8.30 a.m. for the dietary intervention. They were asked to consume the meals within a 10 min period and to avoid physical exertion for three hours afterward, during which time blood glucose determinations were made. Blood glucose concentrations were measured by finger-prick analysis of capillary blood using a HemoCue (HemoCue, Ängelholm, Sweden) blood glucose analyser. Blood samplings were made immediately before consuming the meals (duplicate, baseline), and at 20, 40, 60, 90, 120, 150, and 180 min after the start of food consumption. At 180 min the subjects were asked to rate their hunger, using a visual analogue scale, along the dimension “Not at all” to “Extremely” in response to the question “How hungry are you?”, based on published research on VAS scales for assessing appetite [19].

### 2.10. Data Analysis

Incremental blood glucose responses were calculated by subtracting each individual’s baseline value from subsequent measurements and were then used to determine the incremental area under the curve (IAUC) for each individual by trapezoid summation. The highest postprandial blood glucose peak for each individual, irrespective of the time of occurrence (nearly all were at either 30 or 40 min) was used to determine the mean peak height for each meal (Table 3). Data were entered into an Excel spreadsheet for preliminary analysis. For statistical comparison of means, analysis of variance (ANOVA) was used (GenStat version 11.1, VSNi Ltd., Hemel Hempstead, UK). Breakfasts were described by two factors, kiwifruit (present/absent) and guar gum (present/absent), and these two factors (and their interaction) were tested in the ANOVAs. Participant and week were fitted as blocking factors. Residuals were checked to ensure that the assumptions of ANOVA were met. Least significant differences (LSDs) were used to compare means. A power calculation based on the IAUCs obtained in a comparison of breakfast cereal and kiwifruit-substituted breakfast, showed a sample size of *n* = 17 would be required (*p* = 0.05) in a cross-over design with a power of 80% to detect a difference.

## 3. Results

### 3.1. Analysis of Kiwifruit

Digestive analysis of the kiwifruit provided a value of 9.15 ± 0.34 g/100 mL for total sugars. Consistent with previous analyses of six varieties of kiwifruit, the sugars consisted of approximately equal proportions of glucose and fructose with a minor sucrose component. Therefore the sugar mixture made to substitute for kiwifruit sugars in the kiwifruit-free meals was approximated by a 2:2:1 mixture of glucose, fructose and sucrose.

### 3.2. Digestive Analysis of the Meals

**Figure 2:**
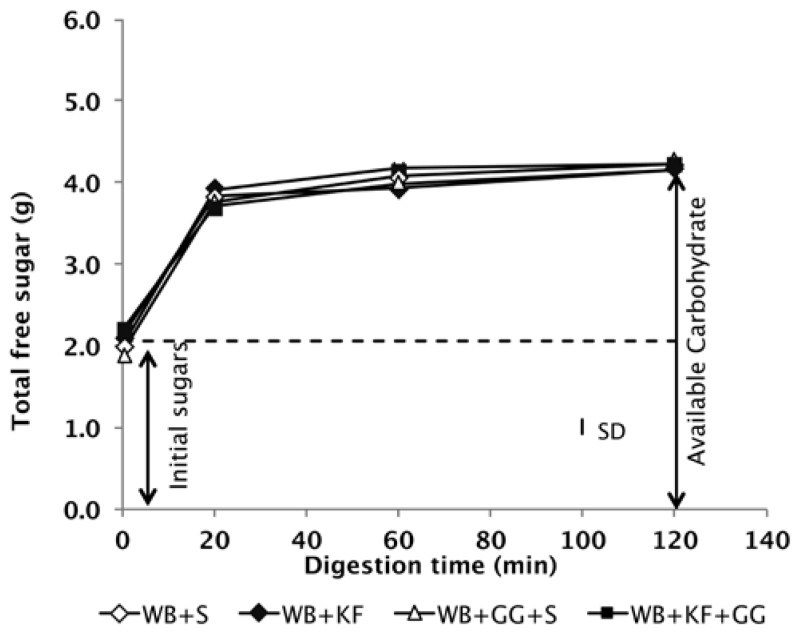
Available carbohydrate content of the meals measured by in vitro digestion. The meals had been formulated to deliver the same quantities of cereal starch and sugars at time of ingestion (S = sugar, WB = wheat biscuit, KF = kiwifruit, GG = guar gum) SD = Mean standard deviation of analyses.

The digestion profiles of the meals during simulated gastrointestinal digestion in vitro confirmed that all four of the meals to be consumed contained the same quantity of available carbohydrate (Figure 2).

### 3.3. Post Hoc Titration of WB+S and WB+KF Meals

**Figure 3:**
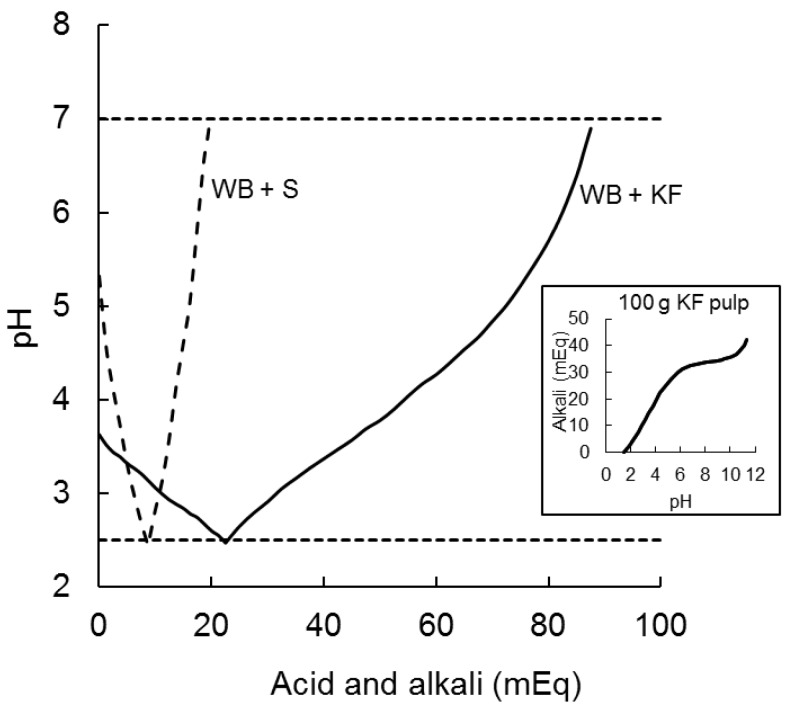
Acid and alkali (milliequivilants) required to reduce the pH of meals ingested from their initial pH to pH 2.5 (with 0.5 M HCl) and to subsequently raise it to pH 7.0 (with 0.5 M NaOH). WB+S (dashed line) was 47.3 g wheat biscuit +20 g sugars; KF+WB (solid line) was 200 g kiwifruit + 47.3 g wheat biscuit. The inset graph shows the typical titration curve of a weak acid with a strong alkali for 100 g kiwifruit pulp, indicating buffering capacity of kiwifruit flesh.

The 200 g of kiwifruit ingested by participants in the present study had a pH of 3.3 which was raised to only pH 3.7 by adding 47.3 g of wheat biscuit in the WB+KF meal. It took 22.5 milliequivalents of acid (0.5 M HCl) to reduce the pH of the KF+WB meaL to pH 2.5, as compared with 8.7 mEq to reduce the pH of WB+S from 5.3 to 2.5. Alkali (0.5 M NaOH) required to subsequently raise the pH from 2.5 to 7.0 were 66 mEq for KF+WB and only 11 mEq for WB+S (Figure 3).

### 3.4. Blood Glucose Responses

#### 3.4.1. Response Amplitude

**Figure 4:**
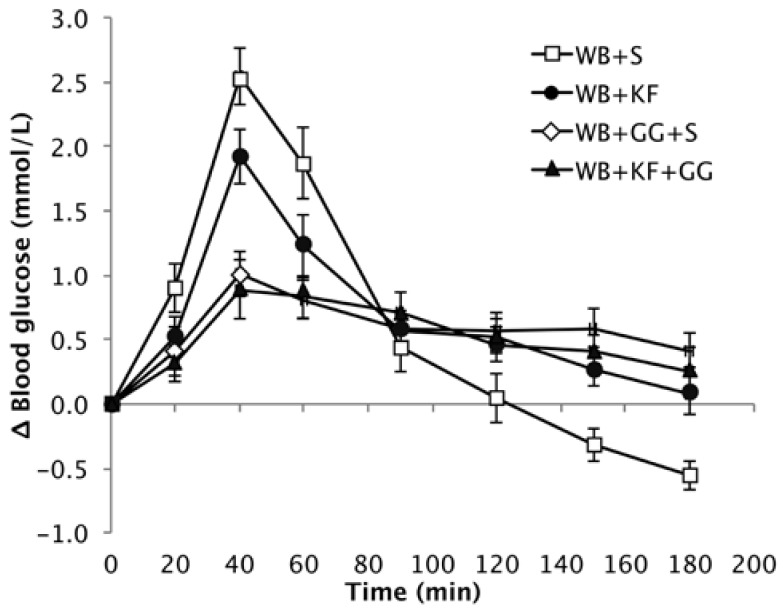
Increase over baseline in blood glucose concentration induced by the meals wheat biscuit (WB)+sugars (S) (WB+S), WB+kiwifruit (WB+KF), WB+guar gum+sugars (WB+GG+S), and WB+KF+GG (Means ± sem).

The baseline values for the different groups were very similar, and the means were not significantly different (Means: 4.5, 4.5, 4.4, 4.6 mmol/L. *p* = 0.471), so the responses are presented as increments over baseline. The different meals induced blood glucose responses that were clearly distinctive (Figure 4).

Peak height showed a very significant difference between the four breakfasts. When comparing the means by LSD (Table 3), the WB+KF induced a significantly lower peak than the WB+S breakfast, but the peaks induced by breakfasts containing guar gum were very significantly lower than induced by both the WB+KF and WB+S breakfasts. That is, GG had a large effect in reducing peak height, KF had a smaller effect on its own, and no extra effect beyond GG when served with GG (GG, *p* < 0.001; KF, *p* = 0.028).

**Figure 5:**
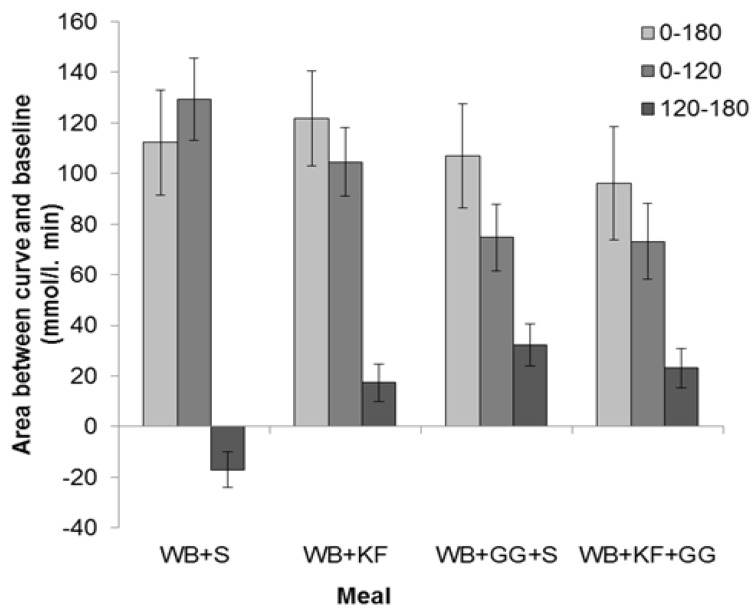
Distribution of incremental areas under the blood glucose response curve in the meals consumed: Wheat bix+sugars (WB+S), WB+kiwifruit (WB+KF), WB+guar gum+sugars (WB+GG+S), and WB+KF+GG. (Means ± sem). LSDs: 0–180 min, 38.9 (*p* = 0.61); 0–120 min, 28.0 (*p* < 0.001); 120–180 min, 16.0 (*p* < 0.001).

The high amplitude postprandial response to WB+S was followed by an overshoot to below the baseline starting at 120 min. In the presence of kiwifruit, the amplitude of the average incremental response was reduced by about 18% (from 7.03 to 6.46 mmol/L). The rate of post-peak decline (mainly blood glucose clearance) was reduced by kiwifruit and/or guar gum, and did not reach the baseline during the three hours of blood glucose measurements. In contrast to the breakfast cereal alone, the meals containing kiwifruit and/or guar were able to sustain a small elevation of blood glucose throughout the entire three-hour measurement period (Figure 4 and Figure 5).

#### 3.4.2. Area between the Blood Glucose Response Curves and Baseline

The IAUC for the meals was calculated for the full 180 min sampling period and for the 0–120 min and 120–180 min sub-periods.

The IAUC over the period 0–180 min showed no significant difference between the four breakfasts based on LSDs (Figure 5) and ANOVA (GG, *p* = 0.266; KF, *p* = 0.969, GG × KF interaction, *p* = 0.462).

The IAUC for the first 120 min, which is the time period recommended for glycaemic index measurements [20] and stipulated in Australian Standard AS 4694-2007 “Glycaemic index of foods”, showed a very significant difference between the four meals (*p* < 0.001). It was greatest for WB+S (129 mmol/L/min), less for KF+WB (105 mmol/L/min), and significantly lower for the meals containing GG (WB+KF+GG, 75 mmol/L/min; WB+GG+S, 73 mmol/L/min). ANOVA, however, confirmed that the only statistically significant effect could be attributed to the GG (GG, *p* < 0.001, KF, *p* = 0.189, GG × KF interaction, *p* = 0.462).

In the period 120–180 min, WB+KF (17 mmol/L/min), WB+GG+S (32 mmol/L/min) and WB+KF+GG (23 mmol/L/min) all gave positive areas between the curve and baseline that differed significantly from the area for the WB+S meal (−17 mmol/L/min), which alone was negative (Figure 5). ANOVA revealed significant effects of both KF and GG, and an interaction (GG, *p* < 0.001, KF, *p* = 0.030, GG × KF, *p* < 0.001)

#### 3.4.3. Blood Glucose at 180 min

**Figure 6:**
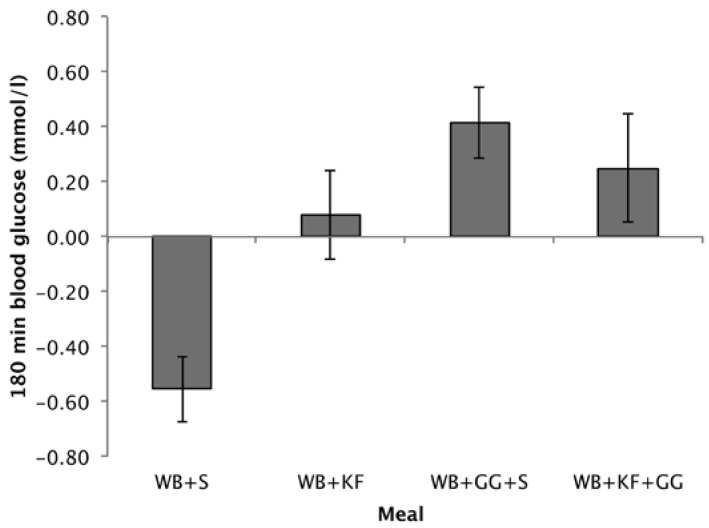
Blood glucose concentrations at 180 min after consuming wheat biscuit+sugars (WB+S), WB+kiwifruit (WB+KF), WB+guar gum+sugars (WB+GG+S), and WB+KF+GG. (Means ± sem; LSD 0.347, *p* < 0.001).

Differences from baseline in blood glucose at 180 min followed a similar pattern to the 120–180 min IAUC. Comparing the means, treatments containing GG gave the highest incremental blood glucose values (WB+GG+S, 0.42 mmol/L; WB+KF+GG, 0.25 mmol/L), the WB+KF was just positive (0.08 mmol/L) but significantly greater than the WB+S breakfast, which was negative (−0.56 mmol/L) (Figure 6). ANOVA revealed significant effects of KF and GG and an interaction (GG, *p* < 0.001, KF, *p* = 0.028, KF × GG, *p* = 0.026).

Whether blood glucose at 180 min was significantly different from baseline was tested using a one-sample t-test comparing the means to zero. After the WB+S breakfast blood glucose at 180 min was significantly lower than baseline (*p* < 0.001). After the WB+GG+S breakfast, it was significantly higher (*p* = 0.005), while for the WB+KF and WB+KF+GG blood glucose was not significantly different from baseline (*p* = 0.629, and *p* = 0.222).

### 3.5. Hunger Experienced

**Figure 7:**
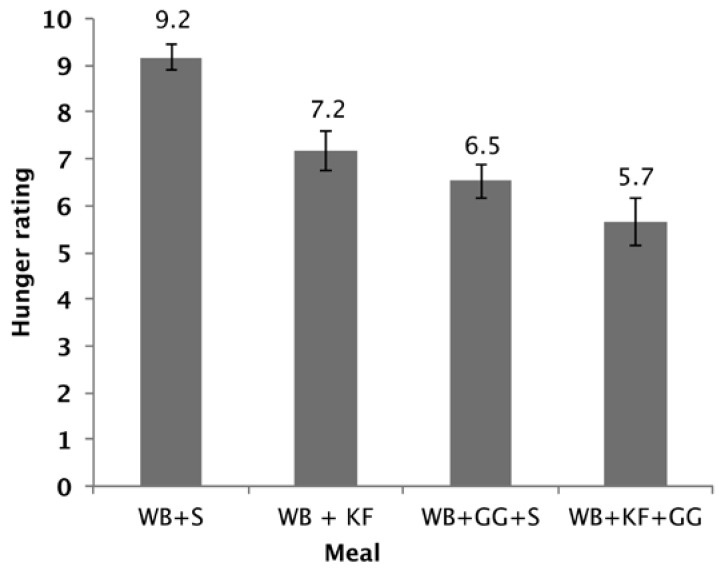
Hunger rating at 180 min after consuming WB+sugars (WB+S), WB+kiwifruit (WB+KF), WB+guar gum+sugars (WB+GG+S), and WB+kiwifruit+guar gum (WB+KF+GG). The subjects were asked to respond to the question “How hungry do you feel?” on a visual analogue scale extending from “Not at all” to “Extremely”, scored on a scale of 1–10. (Means ± sem; LSD 0.96, *p* < 0.001).

The hunger scores showed a significant difference between the four breakfasts (*p* < 0.001). Factorial ANOVA revealed that GG (*p* < 0.001) and KF (*p* < 0.001) both lowered the hunger scores and that there was also a further lowering with the interaction of both factors (*p* = 0.089). Both WB+KF and WB+GG+S breakfasts had significantly lower hunger scores than WB alone, and the WB+KF+GG had a lower hunger score than the WB+KF alone (Figure 7).

## 4. Discussion

As the primary aim of the research reported here was to measure the role of non-carbohydrate factors in any differences between the meals in glycaemic response, it was important to ensure that the meals contained the same quantities of available carbohydrate after formulation. The digestive analysis confirmed that all meals were nearly equal in available carbohydrate and that the available carbohydrate in them did not differ between treatments in intrinsic digestibility, based on the in vitro digestograms (Figure 2).

A secondary objective was to see whether an interaction of kiwifruit with co-consumed hydrocolloid could augment any modulatory effect of kiwifruit on the glycaemic response. The study suggested that kiwifruit components other than sugars might be acting to modulate glycaemic response to co-consumed cereal, with significant effects on response amplitude and response distribution over the 3 h testing period. However, no substantial interactions between kiwifruit and guar were demonstrated, and the response curves for the meals containing guar gum, with or without kiwifruit, were very similar. Therefore, the interaction between kiwifruit remnants and guar gum that resulted in a marked reduction in mixing in vitro [14] was not evident in the present study, probably because of the high concentration of guar used.

The meals containing kiwifruit and/or guar gum improved the glycaemic response profile in two ways—by reducing peak height and by averting the overshoot to below the baseline seen in the WB+S meal. Delays in gastric processing of WB in the WB+KF, WB+GG+S and WB+KF+GG meals may have contributed to the reduced amplitude with a correspondingly less intense glucose disposal so that the glycaemic response was extended and the postprandial overshoot to below baseline avoided. As digestion continues well beyond the immediate postprandial period [21], meals that reduce the rate of digestion may lead to the prolonged slight elevation of blood glucose above the baseline demonstrated in this study, indicating an adequate and sustained supply of glucose from the gut.

Guar gum consumed at the dose used in the present study has been shown to improve cognitive performance, while maintaining blood glucose slightly above baseline. Like guar, kiwifruit alone was able to prevent blood glucose from dropping below the baseline and to significantly reduce hunger at 180 min (Figure 6). This effect may be a consequence of differences in blood glucose combined with physical effects in the gut. Irrespective of the mechanisms involved, the results suggest that if kiwifruit contributes to maintenance of satiety, its effects on appetite are worth investigating further with the fully validated appetite scale in the context of obesity management. Similarly, assuming that satiety reflects physiological state, and given the results obtained with guar [18], the effects of kiwifruit on cognition should be explored in further research. Although the hunger results were possibly limited by experienced hunger not being a complete measure of appetite [19], it correlates with appetite. A possible limitation of the hunger data is that it is a subjective rating, and it was not possible to blind the subjects to the taste of kiwifruit or to the sensation of guar gum in the meals.

The 10 g dose of guar gum used in this study was selected because it had been shown to be effective in suppressing postprandial glycaemic response and to maintain blood glucose concentrations above the baseline for 180 min [18], so can be regarded as a positive control. The concentration of guar in the stomach and intestine would depend on the volume of liquid consumed and on the volume of digestive juices that were produced. A final concentration of 1%, if the guar was dispersed in a volume of 1 L, would be quite viscous [14]. So, it is likely that effects on gastric emptying as well as retardation of digestive processes such as diffusion and mixing contributed to the observed reduction in glycaemic response in the guar-containing treatments. However, a lower concentration may have been required to detect an interaction with kiwifruit in vivo.

A number of specific factors that may have contributed to the overall effect of kiwifruit could be the subject of further research. Remnants of kiwifruit after in vitro digestion, mainly cell wall residues, have been shown to substantially retard gut-level processes involved in the glycaemic response; at physiological concentrations kiwifruit residues reduced glucose diffusion and simulated luminal mixing each by about 50% [6]. Phenolic compounds present in kiwifruit may have inhibited glucose uptake from the gut and/or stimulated glucose disposal in the body [9]. It is also possible that the organic acids of the kiwifruit may have delayed gastric emptying [22] and ileal digestion through the combined effect of them lowering pH of the kiwifruit-containing meal when ingested, and delaying gastric and duodenal pH adjustment through the considerable buffering capacity of kiwifruit demonstrated in the *post hoc* analysis reported here. Therefore, delays in gastrointestinal pH adjustment may have contributed to the retardation of digestive processes involved in the glycaemic response.

The results of the present study have implications for the inclusion of fruit in the diets of people who suffer from glucose intolerance. There are numerous well-established nutritional benefits from consuming fruit, and most nutritional recommendations include the regular daily consumption of fruit, while those with glucose intolerance commonly assume that fruit should be avoided because of their high sugar content. However, the present study has shown that if kiwifruit were to be included in a diet on an equal carbohydrate exchange basis, involving the substitution of readily digested starchy food by kiwifruit, there would be a glycaemic response lowering effect of the non-carbohydrate components of the fruit, as well as an effect of partial replacement of glucose by fructose.

## 5. Conclusions

The present study was designed to show the influence of kiwifruit components other than the available carbohydrates on the glycaemic response, and used meals containing the same quantities of the same carbohydrates to do so. The effects that were noted were worthwhile in terms of improving the glycaemic response profile. Maintaining blood glucose at near baseline and avoiding consequent hunger also has important health implications given the well-established links between energy intake, obesity, and ill-health in a range of forms. However, in terms of incorporating kiwifruit, for its health benefits, into the meals for those who are glucose intolerant and who need to monitor their energy intakes for the control of obesity, it may be necessary to consider using kiwifruit within a carbohydrate exchange regimen to avoid increasing energy intake.

If kiwifruit were to be included in a meal by equi-carbohydrate (approximately iso-energetic) substitution of breakfast cereal, such as WB, one might expect even greater reductions in glycaemic response than were observed in the present study, because the fructose (GI = 22) portion of the carbohydrate in kiwifruit would substitute digestible starch (GI = 70) in a flaked breakfast cereal.

The present results have potentially important implications for health claims related to a role for kiwifruit in diets of populations at risk of diabetes. With a growing concern for the negative impact of increased fructose intakes in fruit and fruit products, it is important to demonstrate that the lowering of glycaemic response by equi-carbohydrate partial exchange of kiwifruit for readily digested starchy foods is due to more than simply substitution of low GI fructose for high GI starch. The design of the present study allowed us to demonstrate that kiwifruit components other than available carbohydrate contribute to the glycaemic response-lowering effect of equal carbohydrate exchange of kiwifruit for starchy staple.

## Figures and Tables

**Table 1:** Weights of meal components used (g).

Meal Component	Meal 1	Meal 2	Meal 3	Meal 4
	WB+S	WB+KF	WB+GG+S	WB+KF+GG
Wheat biscuit (WB)	47.3	47.3	47.3	47.3
Kiwifruit	-	200	-	200
Guar gum	-	-	10	10
Sugar mix ^1^	19	-	19	-
Water ^2^	180 mL ^2^	-	180 mL	-

^1^ Glucose:fructose:sucrose, 2:2:1; ^2^ Approximate water content of 200 g kiwifruit. “–“: absent from meal.

**Table 2:** Available carbohydrate in meals by formulation (g).

Available Carbohydrate Source	Meal 1	Meal 2	Meal 3	Meal 4
	WB+S	WB+KF	WB+GG+S	WB+KF+GG
WB	21.7	21.7	21.7	21.7
Kiwifruit	-	18.3	-	18.3
Glucose	7.32	-	7.32	-
Fructose	7.32	-	7.32	-
Sucrose	3.66	-	3.66	-
Total	40	40	40	40

“–“: absent from meal.

**Table 3:** Peak incremental blood glucose concentrations (mmol L^−1^) in response to consuming meals containing wheat biscuit (WB), kiwifruit (KF) and guar gum (GG).

	Meals				LSD	*p*
	WB+S	WB+KF	WB+GG+S	WB+KF+GG		
Mean	2.72	2.17	1.26	1.26	0.346	<0.001
Sem	0.18	0.21	0.15	0.15		

Sem: Means and standard error of mean.

## Supplementary Materials

The following are available online at http://www.mdpi.com/2072-6643/9/11/1195/s1, Supplementary Table S1: CONSORT checklist.
