# Supplementary material for: Kiwifruit Non-Sugar Components Reduce Glycaemic Response to Co-Ingested Cereal in Humans
Source: Nutrients. 2017 Oct 30;9(11):1195. doi: 10.3390/nu9111195 (PMC5707667; doi:10.3390/nu9111195)
Supplement: Supplementary file 1 [file nutrients-09-01195-s001.docx]

**Supplementary Table S1:**


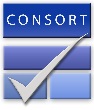
CONSORT checklist

| Section/Topic | |  | Item No. | Checklist Item | Reported on Page No. |
| --- | --- | --- | --- | --- | --- |
|  | Title and Abstract | | | | |
|  | |  | 1a | Identification as a randomised trial in the title |  |
|  |  |  | 1b | Structured summary of trial design, methods, results, and conclusions (for specific guidance see CONSORT for abstracts) | 1 |
|  | Introduction | | | | |
| Background and objectives | |  | 2a | Scientific background and explanation of rationale | 1-2 |
|  |  |  | 2b | Specific objectives or hypotheses | 2 |
|  | Methods | | | | |
| Trial design | |  | 3a | Description of trial design (such as parallel, factorial) including allocation ratio | 5 |
|  |  |  | 3b | Important changes to methods after trial commencement (such as eligibility criteria), with reasons |  |
| Participants | |  | 4a | Eligibility criteria for participants | 5 |
|  |  |  | 4b | Settings and locations where the data were collected |  |
| Interventions | |  | 5 | The interventions for each group with sufficient details to allow replication, including how and when they were actually administered | 7 |
| Outcomes | |  | 6a | Completely defined pre-specified primary and secondary outcome measures, including how and when they were assessed | 5,7 |
|  |  |  | 6b | Any changes to trial outcomes after the trial commenced, with reasons |  |
| Sample size | |  | 7a | How sample size was determined | 7 |
|  |  |  | 7b | When applicable, explanation of any interim analyses and stopping guidelines |  |
| Randomisation: | |  |  |  |  |
| Sequence generation | |  | 8a | Method used to generate the random allocation sequence |  |
|  |  |  | 8b | Type of randomisation; details of any restriction (such as blocking and block size) |  |
| Allocation concealment mechanism | |  | 9 | Mechanism used to implement the random allocation sequence (such as sequentially numbered containers), describing any steps taken to conceal the sequence until interventions were assigned |  |
| Implementation | |  | 10 | Who generated the random allocation sequence, who enrolled participants, and who assigned participants to interventions |  |
| Blinding | |  | 11a | If done, who was blinded after assignment to interventions (for example, participants, care providers, those assessing outcomes) and how | 5 |
|  |  |  | 11b | If relevant, description of the similarity of interventions |  |
| Statistical methods | |  | 12a | Statistical methods used to compare groups for primary and secondary outcomes | 7 |
|  |  |  | 12b | Methods for additional analyses, such as subgroup analyses and adjusted analyses |  |
|  | Results | | | | |
| Participant flow (a diagram is strongly recommended) | |  | 13a | For each group, the numbers of participants who were randomly assigned, received intended treatment, and were analysed for the primary outcome | 6, 7 |
|  |  |  | 13b | For each group, losses and exclusions after randomisation, together with reasons | 7 |
| Recruitment | |  | 14a | Dates defining the periods of recruitment and follow-up |  |
|  |  |  | 14b | Why the trial ended or was stopped |  |
| Baseline data | |  | 15 | A table showing baseline demographic and clinical characteristics for each group |  |
| Numbers analysed | |  | 16 | For each group, number of participants (denominator) included in each analysis and whether the analysis was by original assigned groups | 5-7 |
| Outcomes and estimation | |  | 17a | For each primary and secondary outcome, results for each group, and the estimated effect size and its precision (such as 95% confidence interval) | 7-13 |
|  |  |  | 17b | For binary outcomes, presentation of both absolute and relative effect sizes is recommended |  |
| Ancillary analyses | |  | 18 | Results of any other analyses performed, including subgroup analyses and adjusted analyses, distinguishing pre-specified from exploratory |  |
| Harms | |  | 19 | All important harms or unintended effects in each group (for specific guidance see CONSORT for harms) |  |
|  | Discussion | | | | |
| Limitations | |  | 20 | Trial limitations, addressing sources of potential bias, imprecision, and, if relevant, multiplicity of analyses | 5 |
| Generalisability | |  | 21 | Generalisability (external validity, applicability) of the trial findings |  |
| Interpretation | |  | 22 | Interpretation consistent with results, balancing benefits and harms, and considering other relevant evidence | 13-18 |
|  | Other information | | | |  |
| Registration | |  | 23 | Registration number and name of trial registry | 5 |
| Protocol | |  | 24 | Where the full trial protocol can be accessed, if available |  |
| Funding | |  | 25 | Sources of funding and other support (such as supply of drugs), role of funders | 17 |
